# *Aspergillus flavus* NRRL 3251 Growth, Oxidative Status, and Aflatoxins Production Ability *In Vitro* under Different Illumination Regimes

**DOI:** 10.3390/toxins10120528

**Published:** 2018-12-10

**Authors:** Tihomir Kovač, Bojan Šarkanj, Biljana Crevar, Marija Kovač, Ante Lončarić, Ivica Strelec, Chibundu N. Ezekiel, Michael Sulyok, Rudolf Krska

**Affiliations:** 1 Department of Applied Chemistry and Ecology, Faculty of Food Technology, Josip Juraj Strossmayer University of Osijek, Franje Kuhača 20, Osijek 31000, Croatia; bsarkanj@unin.hr (B.S.); biljana.crevar@ptfos.hr (B.C.); ante.loncaric@ptfos.hr (A.L.); ivica.strelec@ptfos.hr (I.S.); 2 Center for Analytical Chemistry, Department of Agrobiotechnology (IFA-Tulln), University of Natural Resources and Life Sciences Vienna (BOKU), Konrad Lorenzstr. 20, Tulln 3430, Austria; chaugez@gmail.com (C.N.E.); michael.sulyok@boku.ac.at (M.S.); rudolf.krska@boku.ac.at (R.K.); 3 Department of Food technology, University North, Trg dr. Žarka Dolinara 1, Koprivnica 48000, Croatia; 4 Inspecto Ltd., Industrijska zona Nemetin, Vukovarska cesta 239b, Osijek 31000, Croatia; marija.kovac@inspecto.hr; 5 Department of Microbiology, Babcock University, Ilishan Remo 121103, Ogun State, Nigeria; 6 Institute for Global Food Security, School of Biological Sciences, Queen’s University Belfast, University Road, Belfast BT7 1NN, Northern Ireland, UK

**Keywords:** VIS light illumination, *Aspergillus flavus*, mycotoxins, mycelial growth, oxidative status modulation, aflatoxin production, optogenetics

## Abstract

*Aspergillus flavus* is the most important mycotoxin-producing fungus involved in the global episodes of aflatoxin B_1_ contamination of crops at both the pre-harvest and post-harvest stages. However, in order to effectively control aflatoxin contamination in crops using antiaflatoxigenic and/or antifungal compounds, some of which are photosensitive, a proper understanding of the photo-sensitive physiology of potential experimental strains need to be documented. The purpose of the study is therefore to evaluate the effect of visible (VIS) light illumination on growth and conidiation, aflatoxin production ability and modulation of *A. flavus* oxidative status during in vitro experiment. Aflatoxigenic *A. flavus* strain was inoculated in aflatoxin-inducing YES media and incubated under three different VIS illumination regimes during a 168 h growth period at 29 °C. VIS illumination reduced *A. flavus* mycelia biomass yield, both during growth on plates and in liquid media, promoted conidiation and increased the aflatoxin production. Furthermore, aflatoxin production increased with increased reactive oxidative species (ROS) levels at 96 h of growth, confirming illumination-driven oxidative stress modulation activity on *A. flavus* cells.

## 1. Introduction

Contamination of food by mycotoxins represents a global menace affecting economies, trade, and health, with human and livestock health being the most affected. According to Pinotti et al. (2016) [1] the abovementioned overall effects include: crop production losses, losses due to contaminated food and feed disposal, reduction of livestock production, and increasing costs of human and animal health care. Additionally, analytical and regulatory costs are increased, and finally losses of animal and human life occur. These negative impacts of mycotoxins have been suggested to be aggravated by interacting factors of climate change which weigh heavily on major foodborne fungal growth and mycotoxin production potentials [2,3,4,5,6,7]. Out of the over 400 known mycotoxins, aflatoxins are considered the most potent mycotoxins due to their carcinogenic and genotoxic effects in biological systems [8,9].

*Aspergillus flavus*, a saprophytic soil fungus, is the most important producer of mycotoxins including the potent aflatoxins. Consequently, this fungus is involved in crop colonization and contamination with aflatoxins, both during pre-harvest and post-harvest stages of crops. Previous studies have suggested that the dynamics of *A. flavus* growth and its potential to produce toxins are influenced by factors of global climate changes (e.g., temperature, drought stress, and CO_2_ concentration) [10,11,12] while the sensitivity of the fungus to oxidative status perturbations is closely related to the production of aflatoxin B_1_ [13,14,15,16,17,18]. Despite the efforts to control aflatoxin occurrence in food and feed chains worldwide, continuous and increasing contamination levels are being reported in many regions owing to climate change [19,20,21]. Thus, identifying compounds that can decrease contamination will increase the production of safe food, reduce human and animal exposure, and ensure public health. However, some of such compounds can be photosensitive [22] while *A. flavus* has been suggested to be a light-responsive fungus, response varying with fungal strain type [23,24,25]. Aflatoxins have also been reported to be light sensitive compounds [26], giving very dynamic and comprehensive response to the VIS light. 

Prior to further research steps aimed at testing the effect of antiaflatoxigenic and/or antifungal photosensitive compounds on *A. flavus*, the determination of the effects of three different visible (VIS) light illumination regimes (continuous 24 h dark, continuous 24 h VIS, and 12 h dark/12 h VIS) on growth and sporulation, aflatoxin production ability and oxidative status modulation of *A. flavus* during in vitro experiment is required to mimic the natural environment (12 h dark/12 h VIS); thus, the aim of this study. The data obtained from this study will be useful to setup more precise experiments that utilize photosensitive compounds. 

## 2. Results

### 2.1. VIS Light Illumination Period Affects *A. flavus* Growth and Conidiation

**Figure 1:**
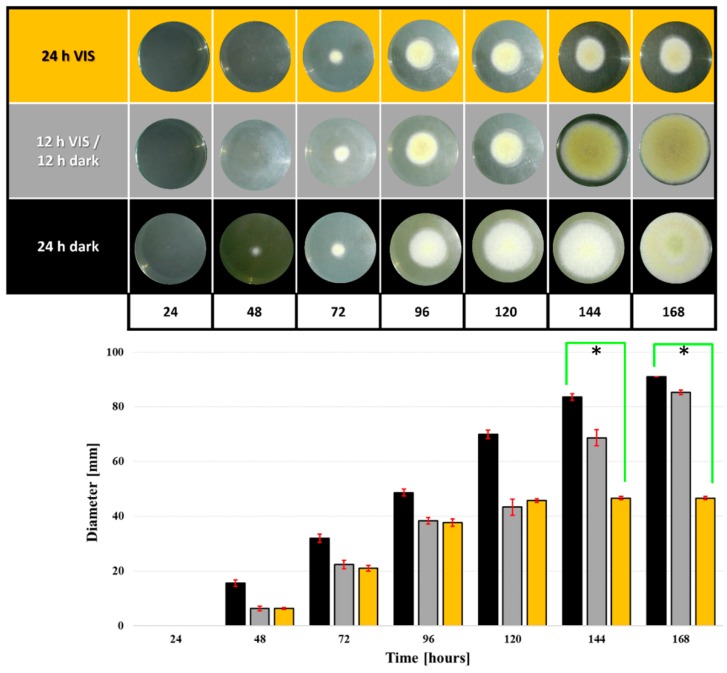
Influence of VIS illumination period on *A. flavus* colony diameter on YEA plates during 168 h growth period at 29 °C. Statistical significance is highlighted with asterisks (*) while data are expressed as mm of diameter (the mean ± SEM) from three separate experiments.

**Figure 2:**
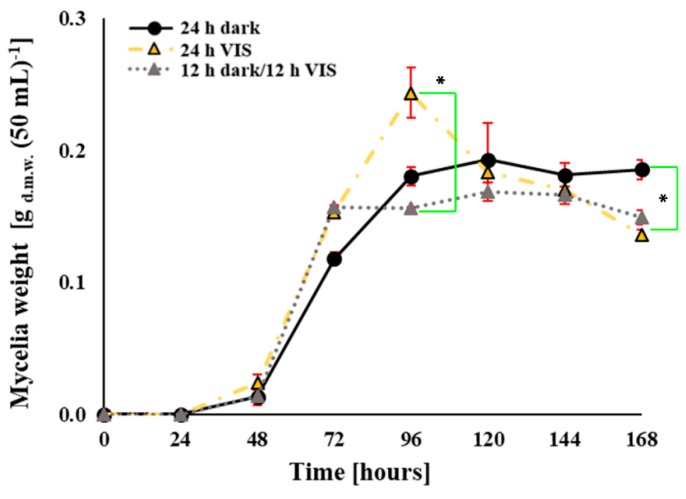
Influence of VIS illumination period on mycelial growth of *A. flavus* (expressed as gram of dry weight (g.d.w.) per 50 mL) in YES medium incubated at 29 °C for 168 h. Statistical significance is highlighted with asterisks (*) while data from three separate experiments are presented by the mean ± SEM.

The influence of VIS illumination period on *A. flavus* mycelial growth was examined on yeast extract sucrose with 2% agar (YEA) plates (Figure 1) and in the YES medium (Figure 2). VIS illumination decreased *A. flavus* growth on YEA plates (Figure 1). Differences in the diameters of fungal colonies were visible at all the time points between growth under 24 h dark and 24 h VIS, although statistically significant differences in colony diameters were only observed at 144 h (*p* = 0.03) and 168 h (*p* = 0.02) time points. Such difference underlies diameter decrease of 44.2% at 144 h and 48.7% at 168 h of growth (Figure 1). Colony diameter growth rates were not different between colonies grown under 24 h dark and 12 h dark/12 h VIS, and between 24 h VIS and 12 h dark/12 h VIS.

Figure 2 presents the influence of VIS illumination period on mycelial growth of *A. flavus* in liquid yeast extract sucrose (YES) medium. The mycelial growth rates of the cultures from 24 h VIS and 12 h dark/12 h VIS did not differ statistically except at the 96th h of growth, where a significant (*p* = 0.03) spike was observed for the 24 h VIS regime compared to the 12 h dark/12 h VIS. The mycelia biomass of cultures grown under 24 h VIS was, however, decreased after that time-point most likely due to oxidative status perturbations. Similarly, statistically significant (*p* = 0.04) difference was recorded for mycelia growth at 168 h of incubation between cultures under the 24 h dark and 12 h dark/12 h VIS periods (*p* = 0.04). Despite this fact, the difference in mycelia biomass yield under 24 h dark regime and the other two variations of illumination regimes significantly decreased by 26.7 (24 VIS) and 19.6% (12 dark/12 VIS), respectively, at 168 h.

**Figure 3:**
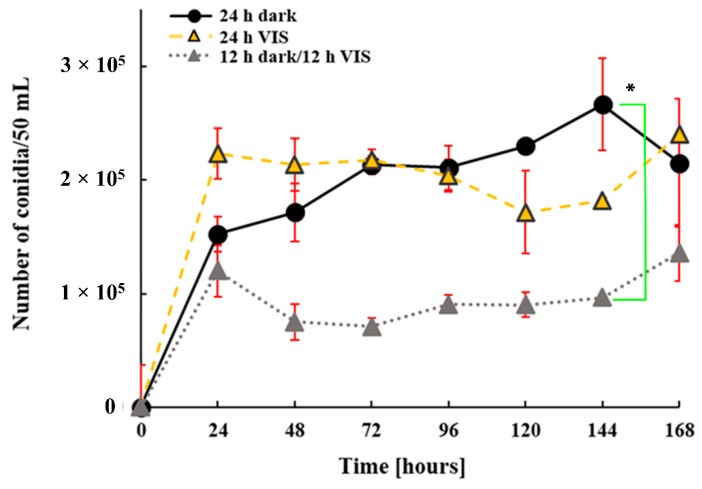
Influence of VIS illumination period on conidia production by *A. flavus* grown at 29 °C for 168 h in YES medium. Data are expressed as conidia number per 50 mL (mean ± SEM) from three separate experiments. Statistical significance is highlighted with asterisks (*).

The impact of VIS illumination period on the ability of *A. flavus* to produce conidia is presented in Figure 3. VIS illumination did not significantly affect the ability for conidia production at the tested regimes, except at 144 h when the conidia number of cultures grown under 12 h dark/12 h VIS illumination significantly (*p* = 0.03) decreased in comparison to those of cultures under 24 h dark. Generally, the number of conidia obtained when 12 h dark/12 h VIS regime was applied was lower by 32.9 to 63.9% between the 48th and 144th h of incubation compared to the conidia counts obtained for the 24 h dark and 24 h VIS at the same time-points.

### 2.2. VIS Light Illumination Period Affects Aflatoxins Production Ability of *A. flavus*

**Figure 4:**
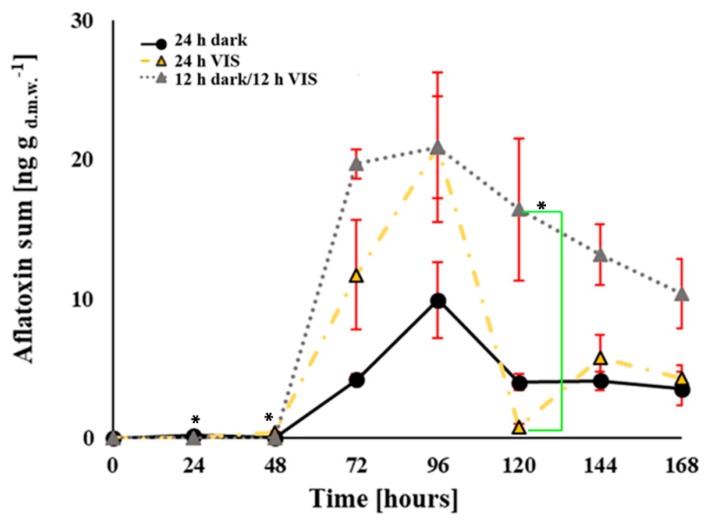
Influence of VIS illumination period on aflatoxin production by *A. flavus* during growth in YES medium at 29 °C for 168 h. Data represents the ng of the sum of produced aflatoxin B_1_ and B_2_ per dry mycelial weight and mL of YES media, and are expressed as the mean ± SEM from three separate experiments. Statistical significance is highlighted with asterisks (*).

**Figure A1:**
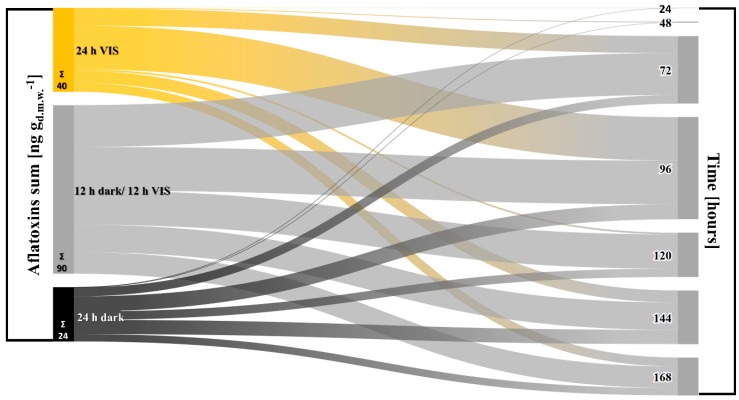
Influence of VIS illumination period on aflatoxin production potential of *A. flavus* grown in YES medium for 168 h at 29 °C. Data represents the ng of the sum of produced aflatoxin B_1_ and B_2_ per dry mycelial weight and mL of YES media (mean ± SEM). Means were calculated from three separate experiments. Legend: Aflatoxin B_1_ and B_2_ sum produced under ■—24 h dark, 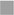
—12 h dark/12 h VIS, 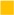
—24 h VIS; Σ—sum of produced concentrations of aflatoxin B_1_ and B_2_.

Analysis of aflatoxin content in YES media showed the dominance of aflatoxin B_1_, while aflatoxin B_2_ contributed on average about 1.19, 1.96 and 2.11% of the sum of aflatoxins produced under 24 h VIS, 24 h dark and 12 h dark/12 h VIS illumination periods, respectively. Therefore, the VIS illumination-mediated aflatoxin (AFB_1_ and AFB_2_) production by *A. flavus* grown in YES medium over a period of 168 h is presented in Figure 4 and Figure A1, and expressed as the sum of the aflatoxin concentrations. Generally, isolates grown under the 12 h dark/12 h VIS regime produced the highest sum of aflatoxin concentrations at all the time points as compared to those grown under the 24 h dark and 24 h VIS regimes, which recorded 67.8 and 50.2% decrease in aflatoxin production, respectively. When the production of aflatoxin B_1_ was examined, statistically significant difference (*p* = 0.02) was observed between an increased toxin level produced at 24 h time point by the culture grown under 24 h dark period (0.02 ng g_d.m.w._^−1^) and a lower toxin level from culture grown under 12 h dark/12 h VIS period (0.00 ng g_d.m.w._^−1^). Furthermore, statistically significant differences were observed for decreased and increased aflatoxin B_1_ production at 48 h (*p* = 0.02) and 120 h (*p* = 0.03) of growth, respectively, under 12 h dark/12 h VIS regime (0.00 and 16.05 ng g_d.m.w._^−1^) in comparison to the toxin concentration produced at same time points under 24 h VIS illumination period (0.11 and 0.22 ng g_d.m.w._^−1^).

### 2.3. Modulation of *A. flavus* Oxidative Status by VIS Light Illumination Period

**Figure 5:**
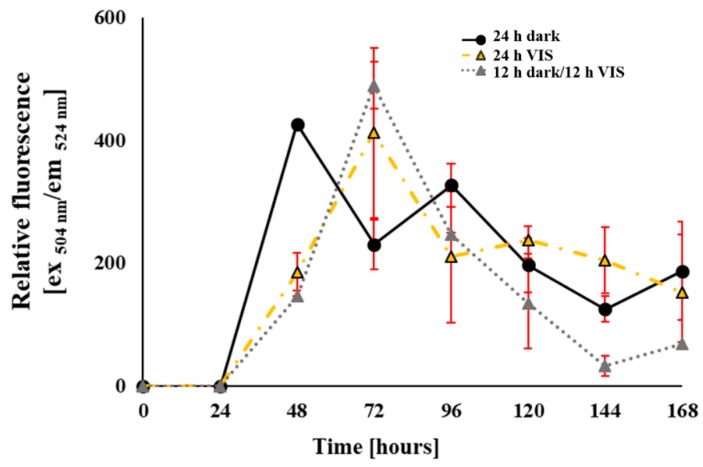
Influence of VIS illumination period on formation of reactive oxidative species in mycelia of *A. flavus* grown in YES medium for 168 h at 29 °C. Data (mean ± SEM) represents relative fluorescence intensity (λ_ex_ = 504 nm, λ_em_ = 524 nm) from three separate experiments.

**Figure 6:**
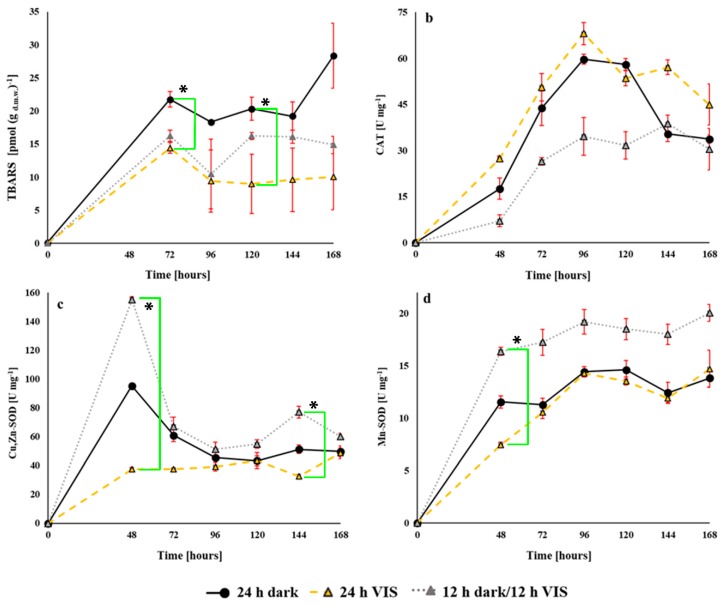
Influence of VIS illumination period on modulation of oxidative status in *A. flavus* mycelia grown at 29 °C for 168 h in YES medium. (**a**) Lipid peroxides are expressed as pmols (mean ± SEM) of thiobarbituric acid reactive substances (TBARS) per dry weight of mycelia and presented by data (mean ± SEM) from three separate experiments. Enzymes: (**b**) catalase (CAT), (**c**) copper, zinc superoxide dismutase (Cu,Zn-SOD), and (**d**) manganese superoxide dismutase (Mn-SOD). Enzyme activities are expressed in U mg^−1^ of protein (mean ± SEM) representing data from three separate experiments. Statistical significance is highlighted with asterisks (*).

Data on the modulation of oxidative status in *A. flavus* by VIS illumination period are presented in Figure 5 and Figure 6. The maximum levels of reactive oxidative species (ROS), measured as relative fluorescence of dichlorofluorescein (DCFH_2_), were recorded at 48 h for *A. flavus* cultures grown under the 24 h dark period and at 72 h for the cultures grown under the other two illumination regimes (Figure 5). Specifically, 60.9% higher ROS content than the ROS values of other two illumination ranges was recorded at the 48 h growth period under the 24 h dark regime, while at 72 h growth period a decreased ROS content of 48.5% was observed in same cultures under the 24 h dark growth regime. After those time points of increased ROS values, ROS values decreased in all tested illumination regimes. Despite the observed trends of ROS values, no statistically significant difference was observed for ROS levels in the *A. flavus* cultures grown under the applied VIS illumination regimes.

In the case of formation of thiobarbituric acid reactive substances (TBARS) (Figure 6a), our results indicate VIS illumination-dependent activity. At all the time points, the highest level of TBARS were formed when *A. flavus* was grown under the 24 h dark regime while the lowest levels were from the cultures grown under 24 h VIS illumination. Statistically significant differences were observed only between cultures grown under 24 h dark and 24 h VIS illumination regimes for 72 (*p* = 0.04) and 120 h (*p* = 0.03) of growth.

Opposite of TBARS formation, catalase (CAT) activity was at the highest rate at all the time intervals in the fungal cultures grown under 24 h VIS illumination region but at the lowest rate when cultures were grown under 12 h dark/12 h VIS (Figure 6b). In general, at every applied illumination regime, after 96 h of fungal growth an inconsistent/staggering trend of CAT activity was observed with a final decrease at 168 h of growth. Specifically, CAT activity of the cultures under the 24 h VIS regime increased to 74.1% at 48 h dropped to 32.3% at 168 h of growth period, in comparison with cultures grown under the 12 h dark/12 h VIS regime. When comparing the cultures grown under the 24 h dark regime and those exposed to 24 VIS illumination, the CAT activity of the latter cultures increased for 35.8% at 48 h of growth and dropped to 25.1% at 168 h. Irrespective of the observed trends, there was no statistically significant difference between tested regimes, except between 24 h VIS period and 12 h dark/12 h VIS at the 48 h time point.

The SOD isoenzymes, copper, zinc superoxide dismutase (Cu,Zn-SOD) (Figure 6c) and manganese superoxide dismutase (Mn-SOD) (Figure 6d) activities of the *A. flavus* cultures grown under the 12 h dark/12 h VIS regime were the highest at all the time points, while these activities were the lowest in cultures grown in the presence of VIS illumination for 24 h.

When Cu,Zn-SOD activities were compared, statistically significant differences were affirmed only at 48 h (*p* = 0.02) and 144 h (*p* = 0.03) of growth of fungal cultures exposed to 24 h VIS illumination and 12 h dark/12 h VIS regimes. At the 48 h growth time point, exposure of the fungal cultures to 12 h dark/12 h VIS illumination caused a 38.6% increased Cu,Zn-SOD activity in comparison to the activity of the cultures grown under the 24 h dark regime. Furthermore, comparing the fungal cultures from the 12 h dark/12 h VIS illumination regime to those from the 24 h VIS illumination period, the increase in Cu,Zn-SOD activity was 75.9% at 48 h and 44.3% at 72 h of growth (Figure 6c).

In the case of Mn-SOD, significant difference was not observed between cultures from 24 h dark period and 24 h VIS illumination, except at the 48 h growth time point when the activity under 24 h darkness increased by 34.4%. Statistically significant difference (*p* = 0.02) was, however, observed at 48 h of growth between Mn-SOD activities of cultures grown under 24 h VIS and 12 h dark/12 h VIS (Figure 6d). The average proportion of Mn-SOD activities in the total SOD activity of the tested mycelia samples were 19.5% for the 24 h dark period, 21.1% for the 12 h dark/12 h VIS, and 23.2% for the 24 h VIS illumination regime.

## 3. Discussion

As already well established, mycotoxin contamination is a reflection of geographical distribution of mycotoxigenic fungi which depends on several environmental factors. Due to predicted climate changes, which are occurring in many parts of the world including Europe, it is of necessity to understand the physiology of mycotoxigenic fungi [19]. The environmental factors that affect fungal physiology are availability of nutrients, pH, environmental temperature, humidity, concentration of CO_2_, and light [10,11,12]. The development of fungi, oxidative stress and secondary metabolite production are integrated sequences of events that determine the relevance of many fungi [16,27,28]. *A. flavus* is the most important mycotoxigenic fungi and the potent carcinogen, aflatoxin B_1_, that it produces is the main cause of different health and economic issues worldwide [29,30,31,32]. This saprotrophic soil fungus infects crops by the dissemination of air-borne conidia or sclerotia on plant debris and in soils, and by the production of high concentrations of aflatoxin B_1_ [23]. Furthermore, the toxicological impact of aflatoxins can be modulated by the combination of other secondary metabolites produced in the environment [33,34].

Consequently, in this study the impact of VIS light, one of the environmental factors that affect fungal physiology, on the growth of mycelia, production of conidia and aflatoxin in *A. flavus* NRRL 3251 in relation to the modulation of cell oxidative status was examined. The focus on impact of VIS light was due to present investigations of new antifungal and/or antiaflatoxigenic compounds, new environmental pollutants, or abiotic stressors which possess certain photosensitive properties. Prior to the impact assessment of the aforementioned compounds on mycotoxigenic fungi under illumination, the impact of light on the tested strain of fungi should first be established.

According to the previous data in literature on the impact of light on various fungal species [23,25,35,36,37,38] and earlier attempted experiments, the changes in mycelia growth rate, conidia production and aflatoxin production ability were expected, while oxidative status of the *A. flavus* cell was modulated. Previous reports from Joffe and Lisker (1969) [39] and Aziz and Mousa (1997) [40], on the impact of light on *A. flavus* were contradictory. However, since these publications were written, much progress has been made regarding insights into environmental factors that affect physiological functions of these fungi. Several publications addressed the exact mechanisms included in regulating the development and secondary metabolism of many *Aspergillus* spp. [23,25,35,36,37,41]. In short, as in many fungal species, the *Aspergillus* spp. development and regulation are directed by *velvet* nuclear complex consisting of global regulatory proteins VeA, Lea and VelB [24,42]. Interestingly, the regulation by VeA is light-dependent, and the result of this regulation are changes in morphology, response to oxidative stress and secondary metabolism. Some studies highlighted the fact that VeA affects the expression of hundreds of genes [36,43]. However, Chang et al. (2012) [44] suggested that VeA light-dependent activities related to vegetative growth, conidiation and biosynthesis of secondary metabolites might vary for different species and strains in *Aspergillus*.

In this study, data confirms that *A. flavus* NRRL 3251 is a VIS light-sensitive fungus, its growth being affected by VIS illumination under the tested regimes. The differences in growth rates on YEA plates and in YES media were expected as observed (Figure 1 and Figure 2). The diffusion of light is at the lowest rate in liquid media due to constant homogenization of media such that the direct impact of VIS light on fungi is reduced. Contrary to that observation, during growth on plates the fungus was exposed directly and constantly to the impact of VIS light. Differences in the diameter of the fungal colonies grown on YEA plates under 24 h dark and 24 h VIS were evident. This was the same observation for mycelia growth rates in YES media under 24 h dark compared to 24 h VIS illumination and 12 h dark/12 h VIS. The findings on growth in YES media under 24 h darkness agrees with our previous findings [45,46,47,48]. However, the mycelia growth rate at 168 h time point contradicts the findings of Azis and Mousa (1997) [40] who stated that increased growth of *A. flavus* was due to illumination impact. However, the previous report of Azis and Mousa (1997) agrees with the increased growth rate under 24 h VIS observed at the 96 h time point. In accordance with data in literature, the rapid increase in fungal growth measured by conidiation and recorded in our study at specific time points under illumination are linked to the sporulation-inducing effect of VIS illumination. Similar finding was established for *A. nidulans* strain where conidiation was promoted by light [24]. In addition, under 24 h VIS, from 24 h growth period until the beginning of idiophase (72 h) the high level photo-induction of conidia also increased, in high level, the oxidative stress of the fungal cells.

It is well established that oxidative stress increases during the early phases (before idiophase) of fungal growth then declines thereafter [28]. This fact was visible from the results obtained for the impact of VIS illumination on aflatoxin production in this study. The production of aflatoxins is triggered by oxidative stress [27]; this was confirmed in this study by the determination of ROS level in the fungal cell. An increase in ROS level at 48 h of incubation under 24 h of darkness caused increased aflatoxin levels at 72 h of growth. The same effect was observed for the other two regimes of illumination; with increase in ROS levels at 72 h of growth, aflatoxin production increased at 96 h of growth. Consequently, our data indicate that an increase in aflatoxin production followed after a period of increased oxidative stress. Similar observation was noted in the case of 24 h VIS illumination where aflatoxin production at 96 h of growth followed after a period of oxidative stress at 72 h of growth. These findings confirm the triggering effect of oxidative stress on aflatoxin production; noting this phenomenon as one of the defense mechanisms of fungal cells [13,14,16,18,28], as well as the fact that VIS illumination modulates oxidative status of fungal cells.

With respect to the enzymatic and non-enzymatic parameters of oxidative stress determined in this study, we report that the oxidative stress of *A. flavus* is increased by VIS illumination at tested regimes (Figure 2, Figure 3, Figure 4 and Figure 5). The TBARS levels increased under 24 h of darkness and combining these with the data for aflatoxins and ROS level it can be concluded our findings are in accordance with the mechanisms proposed by Roze et al. (2011) [41]. They had proposed that increased aflatoxin production under dark conditions could be due to oxidative stress quenching, regulation of conidiation, and protection from inhabitants in the ecological niche (soil which is natural environment of the fungus). Data obtained from the examined enzymatic parameters (CAT, Cu, Zn-SOD, and Mn-SOD) fulfil the hypothesis of modulated oxidative stress due to VIS illumination. The CAT level during fungal incubation under 24 h VIS increased in agreement with the reports of Baidya et al. (2014) [43] who established relation between increased CAT levels and the previously explained *velvet* complex. However, the levels of SOD isoenzymes increased the most in fungal cells incubated under 12 h dark/12 h VIS, while Mn-SOD activity in the total SOD activity increased as the illumination period increased, thus, also confirming the hypothesis of modulated oxidative stress.

In this study, the 24 h darkness regime was a simulation of the growth conditions of the fungus in the soil; this impacted on the examined parameters and ended with increased growth and modulated oxidative status related to increased production of aflatoxins, by signaling pathways described by Roze et al. (2011) [41]. Furthermore, during the other two VIS illumination periods, mycelia growth decreased at 168 h of growth in comparison to growth under 24 h darkness. However, aflatoxin production increased at 168 h of growth due to additional oxidative status perturbations when fungus was grown under 12 h dark/12 h VIS, in comparison to the other illumination regimes. Both observed effects are related to VeA regulation. However, it is more likely that increased aflatoxin production and oxidative status, in case of VIS illumination, were due to *veA* light-dependent activities related to the activation of FluG signalling pathway which includes FluG (a development regulator previously described in other members of *Aspergillus* spp.) [24,49].

In conclusion, the VIS light illumination of *A. flavus* NRRL 3251 during growth reduced mycelia biomass yield, both during growth on plates and in liquid media. Furthermore, VIS light enhanced conidiation and also increased the sum of produced aflatoxins; this agreed with increased ROS levels at 96 h of growth. Increased ROS level confirmed the modulated oxidative status of the test fungus and its role in aflatoxin production.

## 4. Materials and Methods

### 4.1. Chemicals

Yeast extract, potato dextrose agar, agar, and sucrose were purchased from Biolife (Milan, Italy). Ethylenediaminetetraacetic acid disodium salt (EDTA-2Na) was from Pharmacia Biotech (Uppsala, Sweden), stabilized 3% solution of hydrogen peroxide (H_2_O_2_) was obtained from Fluka (Buchs, Switzerland), and potassium cyanide was purchased from Sigma Aldrich (Taufkirchen, Germany). Hydrochloric acid as purchased from Merck (Darmstadt, Germany). Trichloroacetic acid (TCA) and ascorbic acid were from Kemika (Zagreb, Croatia), absolute ethanol was from Panreac (Barcelona, Spain), while butylated hydroxytoluene (BHT) and 2-thiobarbituric acid (TBA) was obtained from Acros Organics (Morris Plains, New Jersey, USA). Superoxide dismutase from bovine erythrocytes (3000 U mg ^−1^ protein) (SOD) and xanthine oxidase from bovine milk (0.4-1.0 U mg ^−1^ protein) (XOD) were purchased from Sigma-Aldrich (Taufkirchen, Germany). Formic acid was purchased from Fluka (Buchs, Switzerland). Aflatoxin standard mix (B_1_, B_2_, G_1_, G_2_) was purchased from Biopure (Tulln, Austria). Acetonitrile and methanol (both HPLC grade) were purchased from J. T. Baker (Radnor, PA, USA).

### 4.2. Cultivation of *A. flavus* on Mycological Media and Conidia Count Determination

*A. flavus* NRRL 3251 culture maintained on malt extract agar (Biolife, Milan, Italy) at 4 °C was used in this study. The *A. flavus* strain was grown on potato dextrose agar (Biolife, Milan, Italy) in the dark at 29 °C for 7 days to stimulate conidia production for the experiment. Yeast extract sucrose (YES) broth was used as liquid medium for *A. flavus* growth, while yeast extract sucrose with 2% agar (YEA) plates were used for *A. flavus* growth on solid culture medium. The preparation of fungal conidia suspension, its inoculation into aflatoxin-inducing YES broth in 250 mL flasks and on YEA plates, as well as incubation were conducted at 29 °C, which favors aflatoxin production, as previously described [46]. Aliquots (5 µL and 20 µL) of spore suspension containing 2.5 × 10^6^ spores were used for inoculation of YEA and YES media, respectively.

The fungal cultures on YEA and YES media were incubated for 168 h and three variations of growth conditions were applied: growth in the dark, under VIS Illumination, and under 12 h dark/12 h VIS illumination. VIS Illumination was performed by two LED lights (50 W, 2250 Lux, Stella). Inoculated YEA plates were photographed every 24 h from the 48th to 168th h of incubation and the diameters of the fungal colonies were measured in perpendicular direction twice per plate. For the inoculated YES media flasks, incubation was performed in a rotary shaker (KS 260 basic, IKA, Staufen im Breisgau, Germany) set at 200 rpm, as previously described [46,47]. Every 24 h from the 48th to 168th h of incubation, samples of media and mycelia were collected from the flasks. Separation of mycelia from the media was performed by filtration. Mycelia samples were stored in 2 mL tubes at −80 °C for 24 h until lyophilisation (Christ, Alpha 1–4 LD, Osterode am Harz, Germany) [48]. Drying conditions were as follows: freezing temperature, −55 °C; temperature of sublimation, −35 to 0 °C; vacuum level, 0.22 mbar. The temperature of isothermal desorption varied from 0 to 22 °C under vacuum of 0.06 mbar. Freeze-drying lasted until constant mass of mycelia was obtained, which was approximately 5 h. Additionally, a portion of the mycelia was taken prior to −80 °C storage and lyophilisation, and oven dried (105 °C for 24 h) until constant mass was achieved in order to determine the dry mycelial weight, while in the portion of media stored at −80 °C the conidia count after separation was performed as previously described by Kovač et al. (2018) [46].

### 4.3. Quantitative Analysis of Aflatoxin Concentrations in Culture Filtrates

A dilute and shoot method previously designed and described by Kovač et al. (2017) [46] was performed for aflatoxin content in culture filtrates estimation. YES media separated from mycelia were filtered through 0.22 µm nylon syringe filter (Labex, Budapest, Hungary), diluted ten-fold with 20% acetonitrile solution in ultrapure water, and 10 µL was injected into a LC-MS/MS system. Separation was performed using an Acquity UPLC H-Class system (Waters, Milford, MA, USA) on Acquity BEH C18 column (2.1 × 100 mm, 1.7 µm) (Waters, Milford, MA, USA). The column was heated to 40 °C, with gradient of eluent A (water with 0.1% formic acid) and eluent B (acetonitrile with 0.1% formic acid). Eluent A was held at 98% for the first 0.5 min, followed by a decrease to 10% at 4.0 min, and it was held for 0.5 min at 10%, followed by an increase to 98% for 4.6 min, and equilibrated for 6 min at a flow rate of 0.5 mL min^−1^. Detection and quantification were performed using a Xevo TQD mass spectrometer (Waters, Milford, MA, USA). Positive mode of electrospray ionization source (ESI) was used, and detection was performed by using the multiple reaction monitoring mode (MRM). Two transitions were monitored for each ion, and all parent ions were in protonated state [M + H]^+^. The MRM transitions (*m*/*z*) for quantification and confirmation of AFB_1_, AFB_2_, AFG_1_, and AFG_2_ were 313 > 285, 315 > 259, 329 > 243, 331 > 313 and 313 > 241, 315 > 287, 329 > 259, 331 > 245, respectively. The capillary voltage was set to 3.5 kV, the source temperature was 150 °C, and the desolvation temperature was set to 400 °C. Desolvation gas flow was maintained at 650 L h^−1^, and cone gas flow was set at 10 L h^−1^. The data were acquired and processed using MassLynx and TargetLynx software (v. 4.1., Waters, Milford, MA, USA). Recovery was assessed by spiking blank YES medium with aflatoxin standard solution at a concentration of 10 ng mL^−1^, and it was 92% for all aflatoxins. Instrumental limits of detection were 0.15 ng/mL, and limits of quantification were 0.5 ng mL^−1^ for all aflatoxins.

### 4.4. Analysis of *A. flavus* Cell Oxidative Status

Extracts of *A. flavus* lyophilized mycelia were used for determination of ROS and lipid peroxides content, as well as antioxidant enzyme activities.

ROS content was determined by the modified spectrofluorimetric method of Davidson et al. (1996) [50]. The oxidation of 2′,7′-dichlorodihydrofluorescein diacetate (DCFH_2_-DA) by ROS was used as fluorogenic probe. Lyophilized mycelia (2 mg) of every examined time-point were incubated with 100 mmol L^−1^ potassium phosphate buffer (pH 5.5) and freshly prepared 10 µmol L^−1^ DCFH_2_-DA. The sample was vigorously mixed on a vortex shaker, and incubated in the dark at 28 ± 1 °C for 15 min. Samples were centrifuged (Centric 150, Tehtnica, Zelezniki, Benedikt, Slovenia) at 2795× *g* for 2 min at 28 ± 1 °C, transferred into cuvettes, and monitored for the presence of fluorescent DCFH_2_ (λ_ex_ = 504 nm, λ_em_ = 524 nm) by Cary Eclipse (Varian, Palo Alto, CA, USA) spectrofluorimeter. The results are expressed as relative fluorescence.

Thiobarbituric acid reactive substances (TBARS) assay was performed according to Luschak and Gospodaryov (2005) [51]. TBARS concentration in mycelia extracts was evaluated spectrophotometrically (Helios γ, ThermoSpectronic, Cambridge, UK) at 535 nm and molar extinction coefficient of malonyl dialdehyde (MDA) (ε_535 nm_ = 156 x 103 L cm^−1^ mol^−1^) was used for the calculation.

Catalase (CAT; EC 1.11.1.6) activities were measured spectrophotometrically according to Reverberi et al. (2005) [27], while slightly modified xanthine/xanthine oxidase/NBT assay according to Angelova et al. (2005) [52] was used for estimation of superoxide dismutase activities (SOD; EC 1.15.1.1) at 505 nm. Activities of two isoenzymes, cyanide sensitive Cu,Zn-SOD and the cyanide resistant Mn-SOD, were estimated. Total SOD activity was measured without, while Mn-SOD in the presence of 8 mM potassium cyanide. Cu,Zn-SOD activity was calculated from the difference between total SOD and Mn-SOD activities.

The values of enzymatic ROS-dependent markers (CAT, Cu,Zn-SOD, and Mn-SOD) of oxidative status were expressed as specific activity, and for this purpose protein concentration in prepared extracts were determined by using of the Bradford assay [53].

### 4.5. Statistical Analysis

Data presented in the study are expressed as the mean value ± SEM of three separately performed experiments. The pooled datasets were checked for normality distribution by Shapiro-Wilk test and compared by nonparametric statistics methods (Friedman ANOVA and Kendall coefficient of concordance; Kruskal-Wallis ANOVA). The software package Statistica 13.3 (TIBCO Software Inc., Palo Alto, CA, USA) was used and differences were considered significant when the *p* value was <0.05. For the drawing of the Sankey diagram Flourish studio was used (Flourish Studio, Kiln Enterprises Ltd., London, UK).
